# Cases of Obscure Peritonitis
*American Journ. April, 1829.


**Published:** 1830-04-01

**Authors:** 


### ALMS HOUSE INFIRMARY OF PHILADELPHIA.

#### CASES OF OBSCURE PERITONITIS.*

Dr. Hugh L. Hodge, one of the physicians to the above Institution, has written a short but sensible paper on those obscurely marked cases of peritonitis occasionally met with in practice, which lead even the ablest into error. In order to put practitioners as much as possible upon their guard, we shall give the main features of the two cases which form the nucleus of his observations.

"*Case* 1. A mulatto man, about thirty years of age, of a delicate constitution. He attributed his illness to exposure to wet and cold; and some days after the commencement of his complaint was brought to the infirmary. He then complained of the usual symptoms of dysentery. His abdomen being however enlarged [tympanitic] and painful, especially on pressure, the patient was treated by means of cathartics, anodynes, and demulcents; the symptoms greatly ameliorated, and he appeared convalescent. In this situation, contrary to advice, he left the infirmary and returned to his usual mode of living. He however was, in a few days, re-admitted into the institution, and again treated as suffering from dysentery. He complained of severe griping pains, irritation of the rectum, tenesmus, with frequent inclination to stool. The discharges per rectum were however peculiar; presenting the appearance of yeast in colour and consistence, as if a large quantity of sero-mucous fluid had been evacuated without any admixture of bile or fæces. The abdomen was tympanitic, and pain was excited by pressure. By the use of alteratives (calomel, ipecacuanha, and opium in combination,) and laxatives, assisted by cold affusions to the abdomen and blisters to the lower extremities, the symptoms of mucous irritation

* American Journ. April, 1829.

were again subdued. The stools became bilious and feculent, and there seemed to be no reason why the patient should not recover. He remained however weak and miserable, was much emaciated, without appetite, had a slight paroxysm of fever in the afternoon; the abdomen was tympanitic, but not painful, except when much pressure was made. In this state he continued with little apparent alteration for several days; his strength however gradually diminished, and he died.

"*On dissection* there were evidently some vestiges of mucous inflammation; but bilious fæces were found in the intestines, and there was nothing in this tissue sufficient to account for the death of the patient. The peritoneal surface however was universally inflamed; lymphatic depositions connected all the viscera of the abdomen; while pure pus was found in circumscribed cavities among the convolutions of the intestines, and the folds of the mesentery. In the latter situation a large abscess was formed. Several portions of the peritoneum were of a dark or livid colour and easily torn, as if in a gangrenous condition."

This a well marked and instructive case of peritonitis supervening, in all probability, on pure dysentery. The progress of the symptoms, and the appearances post mortem seem to prove that such was the order of occurrences, for we do not imagine that on the patient's first admission into the infirmary, peritoneal inflammation existed, at least to any extent. Practitioners should bear in mind the tendency of long-continued, or mismanaged dysentery to give rise to inflammation of the serous membrane, a tendency which we have frequently observed in practice.

*Case* 2. Edward Graham, æt. 30, an Irish labourer of intemperate habits, admitted on the 19th June, 1828, with mania à potu threatening. He was ordered a dose of calomel and jalap which purged him freely, and he appeared convalescent, when he was seized in the evening of the 21st, with sharp pains and swelling in the abdomen, and a frequent desire to go to stool without being able to evacuate any thing. The case being thought to be colic, cal. gr. x., pulv. op. gr. i. were prescribed, and the patient felt better on the 22nd, but the abdomen was still tender and swollen, pulse frequent, full and soft, tongue foul, bowels not opened, *Cups to abdomen—cal.* gr. ij. *pulv. op.* grss. *q. b. h.—enema.* 23d, No better, skin hot; pulse frequent with slight tension; tongue florid at edges, brownish and dry in centre; abdomen swollen and tender: *Cal.* ℈i. *to be followed by oil till bowels act freely. Hirud.* 60 *abdom. fotus.* At 3 P. M. the pain was worse and he was bled to ℥viij, blood cupped and buffy. The bowels were well opened in the evening with much relief; 60 *leeches—senna and salts enema—fomentations.* Next morning he was better the enema having acted well, but at 3 P. M. the abdomen was again tender, and 60 more leeches were applied. On the 25th, the tongue being foul, had cal. gr. x. ext. col. c. gr. x. and an injection in the evening, which opened his bowels, and on the 26th he thought himself so well that he would and did leave the house.

On the 30th, four days after his imprudent conduct, he was re-admitted with acute tenderness on the right side of the umbilicus, below which was an ovoid tumour, *looking* like an abscess, but not *feeling* like one—abdomen swollen—pulse 88, small and tense—tongue clean and florid at edges—cough—frequent slimy stools with griping. *Hirud.* 80 *p. dol.* Next day he was no better; abdomen very tender; pulse quick and tense; tormina and tenesmus. *Cal.* gr. x. *to be followed by oil.* The bowels were freely opened, but the patient on the 2nd was much the same: *Hirud.* 40, *fotus.* In the evening the abdominal tenderness and fever being aggravated he was ordered cold affusions to the belly and cold water injections, with relief. On the 3d he was much the same—*cold water continued—blisters to ancles—ipec.* gr. ¼. *q. b. h.* The blisters excited great irritation, and the patient was much better on the 4th. On the 6th there was little or no tenderness of the abdomen remaining.

Next day the swelling near the umbilicus was pointing, had a tympanitic sound on percussion, and when *kneaded* the air was heard gurgling in the intestine. A blister was applied, the patient began to suffer from diarrhœa, became feeble, and the following is the report on the 20th.

"Has been lingering ever since the 9th without any material change in symptoms. Abdomen until yesterday remained tense and pointed. Dr. Hodge thinks that an abscess has formed in the peritoneum; two blisters have been applied successively without any marked effect; pulse has continued irritable; febrile exacerbations in the evening; small and frequent evacuations from the bowels; tonics, astringents, and opiates have been given without any advantage. Yesterday afternoon the tumour of the abdomen subsided; patient says that he had at the time a considerable discharge of fluid and flatus, but as the stools had been thrown away by the nurse without examination, it could not be ascertained whether or not they contained pus. He is now taking porter and diet to support his strength; all the astringents have failed in checking his bowels; he is now using burnt brandy and galls."

On the 26th an opening formed at the umbilicus through which was discharged about a pint of pure pus. The diarrhœa persisted, cough and debility increased, hectic was fully established, fæco-purulent matter was discharged from the opening at the umbilicus, and death took place on the 1st of August.

*Sectio Cadaveris*. "On opening the abdomen, appearances of extensive chronic peritoneal inflammation were seen. The peritoneum every where presented a leaden hue; pretty firm adhesions had formed between the folds of the intestines, between the omentum and intestines, and between the liver and peritoneum lining the diaphragm, &c.; the omentum and small intestines, just behind the umbilicus, had become agglutinated, and were united at the distance of two or three inches around the umbilicus to the anterior parietes of the abdomen; a complete sac was thus formed which contained pus and feculent matter; the abscess extended as far up as the liver, and had but two openings, one at the umbilicus and another about the size of a crow-quill, into the small gut; the sac was so perfectly formed that no pus was found extravasated amongst the intestines around it; five or six ulcers, about the size of a ten cent piece, were seen on the *external surface* of the small intestines, but *perforating only the peritoneal coat*, except the one communicating with the abscess; a little abscess was also found near the left groin, in the folds of the mesocolon, around which was poured out about a gill of pus. The mucous coat of the stomach showed some marks of chronic inflammation; that of the small intestines was perfectly healthy, showing the ulcers had commenced on the external or peritoneal surface.

The mucous membrane of the large intestine was extensively diseased; looked as if it had been corroded by strong acid, a great portion of it being removed by ulceration; it was of a light chocolate colour, and much thickened."

This is a well marked example of the ravages occasioned by that most intractable malady chronic peritonitis. Of what date were the ulcerations in the mucous membrane in the large intestines? It must be remembered that on the second day after admission when the pain in the belly *first* commenced, there were tormina and tenesmus, symptoms in fact of irritation, if not inflammation, of the membrane in question. The state of affairs about the umbilicus clearly shews that the ulceration which opened into the *small* intestine commenced on the peritoneal surface. We lately witnessed a remarkable instance of the same fact, and no doubts can now be entertained of such being sometimes the case, though the converse is the most frequent.

